# *Mentha*: Nutritional and Health Attributes to Treat Various Ailments Including Cardiovascular Diseases

**DOI:** 10.3390/molecules27196728

**Published:** 2022-10-09

**Authors:** Saddam Saqib, Fazal Ullah, Muhammad Naeem, Muhammad Younas, Asma Ayaz, Sajid Ali, Wajid Zaman

**Affiliations:** 1 Department of Biotechnology, Mohi-ud-Din Islamic University, Nerian Sharif 12080, AJ&K, Pakistan; 2 State Key Laboratory of Grassland Agro-Ecosystems, School of Life Sciences, Lanzhou University, Lanzhou 730000, China; 3 China Sinovita Bioengineering Group, Jinan 250000, China; 4 State Key Laboratory of Biocatalysis and Enzyme Engineering, School of Life Sciences, Hubei University, Wuhan 430062, China; 5 Department of Horticulture and Life Science, Yeungnam University, Gyeongsan 38541, Korea; 6 Department of Life Sciences, Yeungnam University, Gyeongsan 38541, Korea

**Keywords:** phytoconstituents, herbal medicine, antidiabetic, cytotoxic, organoleptic

## Abstract

A poor diet, resulting in malnutrition, is a critical challenge that leads to a variety of metabolic disorders, including obesity, diabetes, and cardiovascular diseases. *Mentha* species are famous as therapeutic herbs and have long served as herbal medicine. Recently, the demand for its products, such as herbal drugs, medicines, and natural herbal formulations, has increased significantly. However, the available literature lacks a thorough overview of *Mentha* phytochemicals’ effects for reducing malnutritional risks against cardiovascular diseases. In this context, we aimed to review the recent advances of *Mentha* phytochemicals and future challenges for reducing malnutritional risks in cardiovascular patients. Current studies indicated that *Mentha* species phytochemicals possess unique antimicrobial, antidiabetic, cytotoxic, and antioxidant potential, which can be used as herbal medicine directly or indirectly (such as food ingredients) and are effective in controlling and curing cardiovascular diseases. The presence of aromatic and flavor compounds of *Mentha* species greatly enhance the nutritional values of the food. Further interdisciplinary investigations are pivotal to explore main volatile compounds, synergistic actions of phytochemicals, organoleptic effects, and stability of *Mentha* sp. phytochemicals.

## 1. Introduction

*Mentha* is a perennial, aromatic, and curative herb which has extensive global distribution. Genus *Mentha* belongs to the family Lamiaceae and comprises 25–30 known species. *Mentha* grows vigorously at low temperatures but could undergo a wide range of environmental conditions. Normally, it can reach a height of 10 to 20 cm or more. This genus emerged from Midland countries and progressively expanded worldwide by either artificial or natural genesis [1]. They are now predominantly found in Eurasia, Australia’ South Africa, and North America. According to various studies, *Mentha* plants have superabundant ingredients of phenolic compounds distinctly phenols, flavonoids, terpenes, quinines, and polysaccharides [2,3]. These phytochemicals paved the way for significant utilization in the production of pharmaceuticals food and beverage industry [1,4,5]. Numerous species of *Mentha* are used as spices and for herbal teas. Generally, every part, for instance, the leaves, stems, and roots of *Mentha*, have been used in tribal and traditional medicines [6,7]. Economically, highly important species are *Mentha aquatica* L. (*M. aquatica*), *Mentha longifolia* L. (*M. longifolia*), *Mentha × piperita* L. (*M. × piperita*), *Mentha spicata* L. (*M. spicata*), and *Mentha arvensis* L. (*M. arvensis*). All these species possess potential phytochemicals, such as iso-menthol, iso-menthone, cineol, limonine, piperitone, carvacrol, dipentene, linalool, thujone, piperitenone oxide, and phellandrene, which play an important role in pharmacy, food, flavor, ointment, and associated industries [1,8,9,10]. The utilization of *Mentha* sp. in the food industry will provide a cost-effective and biocompatible route to control diabetes and obesity [11]. Diabetes is a sort of metabolic disorder accrued due to hyperglycemia with raising of glucose levels in the blood, caused by a lack of insulin or a reduction in the insulin level [12]. The extensive use and economic importance of *Mentha* are due to its nutritional value and ability to replace sugar [6,13,14]. The application of *Mentha* phytoconstituents in food items as preservatives and additives will help to reduce the risk of diabetes and cardiovascular diseases.

The frequency of diabetes and cardiovascular diseases are increasing across the world due to diets consisting of high-fat foods and less exercise [15]. The high amount of triglycerides, flavors, and synthetic preservatives in food reduces food nutritional values and leads to diabetes, obesity, and other chronic diseases [16,17]. It has been reported that 30–80% of people are at risk of diabetes and obesity due to dietary habits and lack of physical activities [18]. Various approaches, such as insulin pills and the utilization of sugar-free food, are adopted to control diabetes and obesity [19,20]. These approaches adversely affect patients’ nutrition status and food enjoyment and severely decline the patient’s quality of normal life. Consequently, it intensifies the utilization of natural products, such as phytoextracts and essential oils, to boost the nutritional values of food and reduce the risk of diabetes and obesity [21,22]. In the last two decades, continuous efforts have been made to control metabolic disorders via natural routes, such as ingestion of dietary products. Several chemical drugs are used in food processing, but research has revealed adverse side effects, encouraging the use of active natural compounds [23,24,25,26]. Plant-derived extracts, in pure form or adulterated form, provide endless opportunities as healthy and biocompatible food products [27,28]. Currently, epidemiological researchers suggested many medicinal and aromatic plants for their nutritional and preservative abilities [29,30]. The aqueous extracts of medicinal plants can be used in dietary products to provide plant-based food nutrition to human beings [31,32]. Aqueous extracts are usually obtained from the aqueous phase through a physical process that does not influence their composition [33]. However, prior to the use of these extracts at mass scale, thorough investigations, such as cytotoxicity, antioxidant, antidiabetic activities, and lipid oxidation potential, are necessary to ensure their efficacy and safety through proof-of-concept research for potential health claims [34,35]. *Mentha* is a medicinal and economically important plant that is regularly used for the treatment of vomiting and nausea, its antiallergic effects, its antifungal and antibacterial effects, its antidiabetic effects, the treatment of obesity, the treatment of gastrointestinal diseases, its anticarcinogenic effects, and pain relief [1,36,37].

We compiled the main characteristics of genus *Mentha* extracts which should be considered as food additives and preservatives to help in diabetes and obesity control due to synthetic food additives and preservatives. In addition, we highlighted the challenges, techniques, and opportunities to improve flavor and textural properties to maintain the needs of taste and aroma of the individual. We concluded that the genus *Mentha* species possess potential phytochemicals and flavoring agents, which can be used in daily diet products to improve food quality cost-effectively and sustainably. Furthermore, an investigation of other medicinal and aromatic plants should be considered, specifically their potential as food additives and dietary supplements and ability to control lethal disorders such as diabetes and obesity via daily dietary products.

## 2. Genus *Mentha*: Morphology and Systematics

### 2.1. Morphology

**Figure 1:**
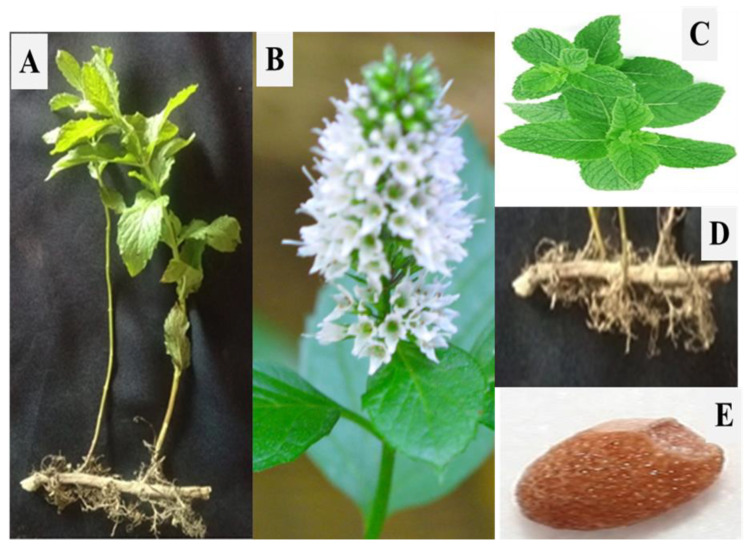
Morphology of *Mentha arvensis* L. (**A**) Shoot structure; (**B**) Flower; (**C**) Leaves; (**D**) Rhizome; (**E**) Seed.

*Mentha* L. is a perennial herb, spread through long slender rhizomes. The rhizomes spread rapidly, and consequently, various populations of this species comprise a progression of clones. The rhizomes sections spread especially along wetlands and riverbanks, resulting in vegetative multiplication and dispersal [38]. The plant has broad ovate leaves rounded or sometimes lanceolate at the base with pubescents and thick-veined leaves (Figure 1). The flowers are arranged in a large whorl with a triangular teeth calyx, and anthers exerting from the corolla. The flowers are mostly protandrous, and usually, self-pollination occurs [1,38].

### 2.2. Systematics

*Mentha* was depicted by Carl Linnaeous from a plant specimen collected from Sweden, who named it *M. canadensis* L. Bentham pursued Linnaeous in keeping *M. canadensis* L. as a subglabrous assortment (var. glubrata Benth.) and a villose one (var. villosaBenth.) [39]. However, recent information based on physiological, anatomical, and molecular attributes have demonstrated that *Mentha* can be grouped into 42 species, hundreds of subspecies, varieties, and cultivars, and 15 hybrids [40]. The scientific classification of *Mentha* is exceptionally unpredictable and there is no consensus. *Mentha* is generally classified into five sections, i.e., *Eriodontes*, *Mentha*, *Preslia*, *Audibertia*, and *Pulegium* [41]. Recently, Zahra et al. [42] reported that phylogenetically, *M. arvensis*, *M. spicata*, and *M. × piperita* show 98% identity when using *matK* sequencing.

## 3. Essential Oil and the Chemical Composition of the Studied Species of *Mentha*

In a true sense, essential oils are not really oils; they are in fact volatile chemicals, produced by living organisms, and are mostly extracted by distillation [43,44]. *Mentha* species contain essential oils with different chemical compositions; for example, in *M. pulegium* L., natural compounds have been reported to account for 96.9% of the chemical profile, including oxygenated monoterpenes, monoterpenes hydrocarbons, oxygenated sesquiterpenes, and non-terpene hydrocarbons. The essential oils separated from leaves of *M. pulegium* contain carvone (56.1%), limonene (15.1%,) E-caryophyllene (3.6%,), oleic acid (3.2%), and 1,8-cineole (2.4%) [45]. Variations in the essential oil composition and its chemical composition were also observed in some species of *Mentha*. Major compounds in *M. × piperita* were observed, including 1-menthone, isomenthone, menthol, menthyl acetate, caryophyllene, and germacrene-D. The study reported a sufficient amount of oil composition, varying from 0.63% germacrene-D to 51% menthol. This indicates that *Mentha* species contain menthol in maximum quantity [46]. Therefore, the plant has the potential to be used as a medicinal ingredient in the food industry to reduce the risk of cardiovascular diseases. The same study reported 12 essential oil compounds in *M. longifolia* with different concentrations of oil compounds from April to July. Another study reported pulegone (86.64%) as a major constituent from *M. pulegium*, possessing antioxidant, quorum sensing, antiinflammatory and antimicrobial activities, indicating that the plant has the potential to reduce the risk of cardiovascular diseases [46]. The chemical composition of Peppermint oil was reported to include oxygen-containing substances, such as menthone (20%), menthol (45–50%), and sesquiterpenes about 3% [47]. It has been reported that *M. spicata* contains major essential oil compounds, including oxygenated monoterpenes (approximately 67%), sesquiterpenes hydrocarbons (7.5%), monoterpene hydrocarbons (approximately 20%), oxygenated sesquiterpenes (1.2%), and other compounds (1.7%) [47]. Piperitrone (81.18%) and piperitenone oxide (94.8%) were also reported from *M. spicata* [47]. Detailed information of the essential oils and its composition is provided in Table 1 of some common *Mentha* species (Table 1). The presence of essential oils indicate that *Mentha* exhibit high antioxidant, antiinflammatory, and antimicrobial potential, which would help to control the risk of cardiovascular diseases by using *Mentha* species compounds in food products [48,49].

The essential oils of *Mentha* are using in aromatherapy. Many food and beverages industries are using *Mentha* as food additive and flavoring agent. Due to aromatic compounds and secondary metabolites, fresh or dried leaves of *Mentha* are used in chewing tobacco, confectionaries, analgesic balm, perfumes, candies, and the tobacco industry [66]. Some researchers found potential antidiabetic effects of *Mentha* [67,68]. The use of *Mentha* in food industry will open new avenues for epidemiologists to control diabetes and cardiovascular diseases.

## 4. Health Benefits of *Mentha*

**Figure 2:**
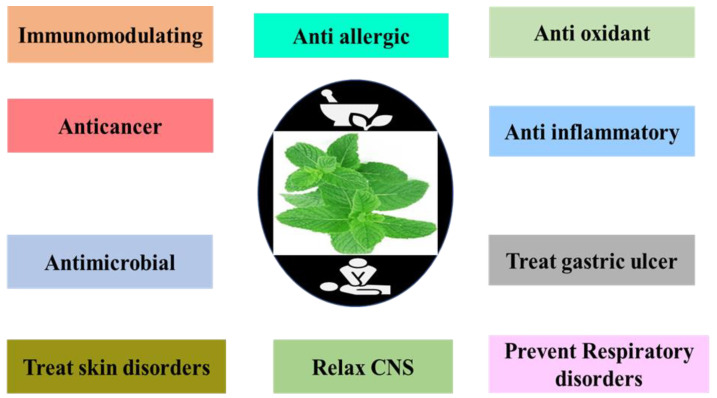
Traditional therapeutic uses of some species of *Mentha* against a variety of ailments.

*Mentha* is a much desired and demanded herb due to its medicinal and therapeutic use. The use of *Mentha* species has been reported in China since the rule of Ming [69]. *Mentha* became an official item of Materia medical in London Pharmacopeia [70]. In the 18th century, it was commonly used as a medicinal herb [71,72]. Various health benefits of *Mentha* species have been reported [50,64]. *Mentha* species have shown analgesic activity during in vivo experiments on mice [61]. *Mentha* species showed antibacterial and antifungal activities against different bacterial and fungal strains [73]. *Mentha* species have traditionally used against various diseases and have the potential to be used for cardiovascular diseases [68]. Several studies have indicated that *Mentha* species contain free radical species and nonradical species, e.g., hydrogen peroxide, which is harmful for molecules of microbes, such as proteins, lipids, nucleic acids, and carbohydrates. Extracts and essential oils of *Mentha* species have shown several health benefits (Figure 2) [74,75].

Some studies found that mint enable lungs surfactants to filter the air and perform better pulmonary action. Methanol from the mint stimulates respiratory muscle strength and increases the end tidal oxygen rate in the human body [76,77]. *Mentha* plants contain constituents with cytotoxic properties, and could be used in developing anticancer agents; for example, *M. longifolia*, *M. arvensis*, and *M*. *× piperita* were found to possess cytotoxic activity against breast cancer in humans [78,79] and human laryngeal epidermoid carcinoma [80]. The direct application of *Mentha* on the skin shows excellent analgesic activity, producing a cooling effect on the skin. Mint oil stimulates blood receptors on the skin and expands blood vessels, resulting in a cold sensation and relaxation [69]. *Mentha* sp. possesses various secondary metabolites which are useful against different disorders (Table 1). These can be used in the food industry to reduce malnutritional risks in diabetic and cardiovascular patients.

## 5. Biological Activities of *Mentha*

A detailed survey of the biological activities of *Mentha* is a prerequisite to explore its potential for the treatment of diseases.

### 5.1. Antimicrobial Activity of Mentha

*Mentha* exhibits a strong antimicrobial potential, which is why it is considered as one of the most industrially, medicinally, and economically important plant genera. *Mentha* has shown a significant antibacterial resistance against the epidemic bacterium Chlamydia. Additionally, *Mentha* helps fight pneumoniae associated with respiratory disease [81]. A study conducted by Hussain et al. [82] reported a strong antibacterial potential of various *Mentha* species. Another study found *Mentha* extracts to have an effective inhibition activity against various strains of bacteria, including *Pseudomonas aeruginosa*, *Shigella flexineri*, *Klebsellia pneumoniae*, and *Styphylococcus aureus* (do Nascimento, Rodrigues, Campos, & da Costa, 2009). Mimica-Dukić et al. [83] isolated secondary metabolites from *Mentha* and tested them against *Escherichia coli* and *Shigella sonei*; they showed significant antibacterial activity. Furthermore, using *Candida albicans* and *Trychophython tonsurans*, studies have shown that *Mentha* extracts have strong antifungal properties. Another study by López et al. [84] reported the potential of *Mentha* extracts against *Rhizopus tolonifera*. Apart from this, various species of *Mentha* have been shown to possess potential antimicrobial activity against resistant pathogens (Table 2), indicating that metabolites of *Mentha* species are highly active against pathogenic organisms. The antimicrobial mechanism of *Mentha* extracts involve the production of antioxidant agents which disrupt the microbial membrane, and subsequently, damage the cellular organelles. The strong antimicrobial potential of *Mentha* extracts proved it as a highly essential preservative in the food industry. Further studies are required to find which kinds of extracts and which elements are important for the production of health-oriented food.

### 5.2. Antioxidant Activity of Mentha

The antioxidant activity of the plants and its extracts is of great importance in fundamental science and applied science. Various species of *Mentha* have shown significant antioxidant activity both in vitro and in vivo. One study reported on the antioxidant activity of *M. longifolia* oil, with an IC_50_ value of 0.659 mL/mL of solution [93]. The antioxidant activity of the five *Mentha* species, including *M. longifolia*, *M*. *× piperita*, *M. spicata*, *M*. *rotundifolia*, and *M. pulegium*, was tested by diphenylpicrylhydrazyl (DPPH) and 2,2′-azinobis (3 ethylbenzothiazoline 6 sulfunic acid) radical (ABTS0+). This study revealed that *M*. *× piperita* exhibits the most strongest DPPH scavenging activity [94]. The methanolic extracts of six *Mentha* species, which included *M*. *villosa* Huds., *M. arvensis*, *M. pulegium*, *M*. *× piperita*, *M*. *rotundifolia* (L) Huds., and *M. aquatica*, were tested. The overall results showed extraordinary antioxidant activity, but *M. aquatica* showed the highest antioxidant potential, with an IC_50_ value of 7.50 μg/mL [95]. Ethanolic extract of *M. pulegium* improved the catalase, glutathione, and peroxidase level after induced toxicity of CCI4 intraperitoneal injection in rats [96]. Another study reported on the antioxidant potential of *M*. *× piperita* by examining various extracts, such as chloroform, ethanol, and aqueous and essential oils, showing 73 to 91% antioxidant capacity at 734 nm and 70.3 to 92.6% free radical scavenging activity [66]. These findings suggest that species of *Mentha* exhibit significant antioxidant potential, and therefore, they are ideal sources for the medicine and food industry to fight cardiovascular diseases.

### 5.3. Antidiabetic Activity of Mentha

Diabetes is one of the main factors of cardiovascular diseases. Therefore, potential resources of a natural origin are required to help in the reduction of diabetic and cardiovascular diseases. *Mentha* oils and extracts exhibit a strong antidiabetic potential, as reported by several researchers. The essential oil of *M*. *virdis* was assessed by the inhibition of α-glucosidase and α-amylase. The results showed that essential oils of *M*. *virdis* exhibit IC_50_ = 101.72 ± 1.86 μg/mL inhibitory potential against α-amylase and IC_50_ = 86.93 ± 2.43 μg/mL against α-glucosidase [3]. Antidiabetic activity of *M. arvensis* L. was determined by in vitro and in vivo experiments in rats. The methanolic extract of *M. arvensis* revealed more than 50% α-amylase and more than 68% α-glucosidase inhibition. Additionally, in rats, significant postprandial hyperglycemia inhibition was observed [97]. Essential oils of *M*. *suaveolens* were found to be very active against α-glucosidase and α-amylase, indicating an inhibitory potential of IC_50_ 141.16  ±  0.2 and 94.30  ±  0.06 μg/mL, respectively [64]. Bayani et al. [98] reported on the antidiabetic effect of aqueous extract prepared from *M. spicata* leaves. The LD_50_ of the extract was more than 1500 mg/kg. The application of the extract showed a significant reduction in cholesterol, low density lipoprotein cholesterol, and triglyceride in diabetic rats as compared to commercially available antidiabetic drug (glebenclamide), indicating that the plant extract possesses a high antidiabetic activity. Thus, it is clear that the use *Mentha* species directly or indirectly can help to reduce the risk of diabetes, and ultimately, reduce the risk of cardiovascular diseases. Based on the literature review, further research is required to screen *Mentha* sp. against specific diseases. Additionally, the search for medicinally important compounds in *Mentha* extracts is also necessary if *Mentha* is to be used as a source of producing and preserving health-oriented food to control diabetes and cardiovascular diseases.

## 6. Cardioprotective Potential of *Mentha* by Its Antiinflammatory Effect

The *Mentha* species that exhibit effective antioxidant compounds (Polyphenolic) play an important role in reducing the risk of cardiovascular diseases by the suppression of inflammation. One of the species of *Mentha* genus, *M. arvensis*, has shown a cardioprotective potential via inhibition of inflammation [99]. Another species, *M*. *× piperita*, revealed antiinflammatory activity against chronic and acute inflammation [100]. The mechanism involves suppression of tumor necrosis factor-alpha (TNF-α), fibroblast growth factor-2 (FGF-2), and vascular endothelial growth factor (VEGF) [101]. As cardiovascular patients have high inflammation, the inflammatory activity of *M. × piperita* may be responsible for reducing risks of cardiovascular diseases. The cardiovascular effects of *M. × piperita* were also reported by Badal et al. [102]. Another species of *Mentha* genus, *M. pulegium*, plays role in the reduction of IL-6, TNF-α, and MCP-1 secretion in murine RAW 264.7 macrophages [103,104]. Moreover, other biological properties, such as the antioxidant, cytotoxic, antidiabetic, and antimicrobial potential of *Mentha*, also improve the cardioprotective potential of *Mentha*. Adding *Mentha* compounds and extracts in food products can facilitate the design of functional foods possessing beneficial health effects.

### Mechanism of Active Compounds with Cardioprotective Effects

**Figure 3:**
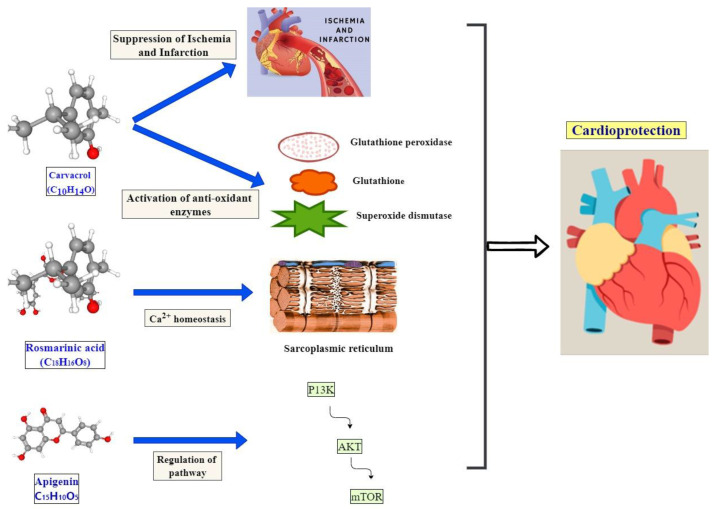
Mode of action of phytochemicals in cardioprotection. This figure was created with https://app.diagrams.net/ (accessed on 15 August 2022).

*Mentha* plants possess a variety of bioactive compounds with cardioprotective and other medicinal properties, among them carvacrol, rosmarinic acid, quercetin, baicalein, and apigenin. These compounds have cardioprotective effects by regulating numerous molecules, such as growth factors, enzymes, kinases, inflammatory molecules, transcriptional factors, apoptosis, etc. (Figure 3). Menthol from *M. arvensis* exhibits activity against ischemic heart disease [105]. Phenolic compound quercetin extracted from the leaves of *M. pulegium* was reported to have cardioprotective effects [106]. Similarly, Pulegone and menthofuran isolated from *M. longifolia* and *M. aquatica* possess antiinflammatory effects, which eventually help in reducing the risk of different diseases in the body [107]. Studies also revealed various functions of *Mentha* species exhibiting cardioprotective effects, decreased toxicity, antiarrhythmic effects, heart rate normalization, and antihypersensitive effects (Table 3) [108,109,110,111].

Carvacrol is a phytochemical, also reported from *Mentha longifolia*, which exhibits a cardioprotective effect through various mechanisms. It suppresses the myocardial ischemic damage in rats of acute myocardial infarction. The compound reduces the infarct size and myocardial enzymes, such as lactate dehydrogenase, creatine kinase, and cardiac troponin T [105]. Carvacrol also increases activities of antioxidant enzymes, including glutathione peroxidase, glutathione, and superoxide dismutase, and reduces malondialdehydes, which facilitate heart protection from cardiac disorders. Carvacrol also promotes the activation of the Akt/eNOS pathway in cardiomyocytes, helping in cardioprotection [112].

Rosmarinic acid shows cardioprotection effects through the regulation of antioxidant enzymes and gene expression of sarcoplasmic reticulum Ca^2+^ ATPase 2 (SERCA_2_) and ryandodine receptor-2 (RyR_2_), which play a role in Ca^2+^ homeostasis [113]. Another study revealed that rosmarinic acid provides protection against cardiac fibrosis through the regulation of AMPKα, nuclear translocation of Smad3, and suppression of phosphorylation. It also induces peroxisome proliferator-activated receptors (PPAR-γ) to constrict cardiac fibrosis [114]. Apigenin present in *Mentha* tissues facilitate cardiac protection by regulating PI3K/AKT/mTOR pathway and inhibited adriamycin-induced cardiotoxicity in rats [115]. Quercetin also showed in vitro and in vivo cardioprotection activities. It inhibits MAPK and focal adhesion kinase activities regulated by thrombin in endothelial cells, leading to cardioprotection [116]. Baicalein promotes the downregulation of the phosphorylation of Ca^2+^/calmodulin-dependent protein kinase II (CaMKII) with the expression of Na^+^/Ca^2+^ exchangers (NCX1), which leads to protection from cardiovascular disorders [117]. *Mentha* species possess numerous bioactive compounds that facilitate cardioprotection from lethal disorders (Table 2). The isolation and applications of these bioactive compounds can facilitate the food industry in the production of functional foods that can protect from cardiovascular disorders.

## 7. Utility of *Mentha* in Food Industry

*Mentha* is a widely cultivated crop, and several species are used in industry. Currently, phytochemicals of *Mentha* plants are used to improve the flavor in food beverages. Mint has different flavors and is the third most demanded flavor on the world food market [123].

*Mentha* oils are commonly connected with flavors used in chewing gums and dental pastes; however, they have many other flavors, which are used in the food industry, ranging from candies, dairy products, sauces, and alcoholic and nonalcoholic beverages [124]. *Mentha* plants are also used to preserve, improve the quality, and extend the life of food.

**Figure 4:**
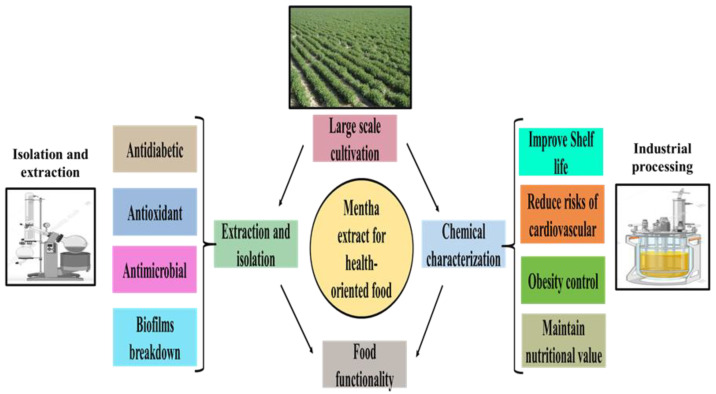
Large-scale cultivation, isolation, and characterizations of *Mentha* phytoconstituents to reduce the malnutritional risks of cardiovascular diseases. This figure was created with Microsoft PowerPoint (accessed on 15 August 2022).

The peppermint flavor of *Mentha* is basically menthol. Menthol causes a cooling effect in the oral cavity and activates cold-sensitive receptors [125]. This molecule also has a sensation of bitterness, so it stimulates both taste and aroma receptors. It releases its minty flavor to food products and other daily life essentials, e.g., tooth paste and mouth fresheners, causing a physiological cooling effect [126]. The essential oils of *Mentha* are used in aromatherapy. Many food and beverages industries use fermented *Mentha* as a flavoring agent. Due to aromatic compounds and secondary metabolites, fresh or dried leaves of *Mentha* are used in the chewing tobacco, confectionaries, analgesic balm, perfumes, candies, and tobacco industries [127]. Large-scale cultivation, isolation, and characterizations can facilitate the food industry to utilize *Mentha* extracts for different purposes (Figure 4). However, there are still significant knowledge gaps, especially regarding the differing potential of the various composition extracts of different plants; these gaps should be filled to ensure cost effective, compatible ways for the production of foods that include *Mentha*. It is important to compare extracts of *Mentha* with other aromatic and medicinal plant extracts, in order to determine which plant extracts are significant for the herbal medicine industry and nutraceutical industries.

## 8. Current Challenges and Implementations

Many mint derivatives and their active compounds have been approved by the European commission and the United States Food and Drug Administration for their proposed used as flavoring agents in food products. Plant extracts have numerous intrinsic and extrinsic challenges, which has hindered their applications in the food industry [128,129]. The exiguity of raw materials, chemotypic diversity, inconsistent efficacy, unexplored molecular mechanism of action, adverse effects on food taste, low water solubility, high cost, and threat to biodiversity loss are some leading challenges to *Mentha* use in the food industry [130]. Moreover, plant collection and identification are difficult due to the close resemblance of different *Mentha* species, in addition to the deficiency in the quality assessment of raw materials. Moreover, there is a scarcity in the quantity of extracts from the raw materials of *Mentha* for industrial applications. After mixing with a food matrix, i.e., fat, protein, carbohydrates, salt contents, pH, moisture, etc., together with extrinsic factors (temperature, gaseous composition, and microbial diversity), the antimicrobial potential of *Mentha* extracts is reduced [131]. The excessive aroma present in plant extracts may negatively influence the organoleptic properties (flavor, color, taste, and texture) of food items, leading to a reduction in consumer demand [132]. Due to the abovementioned challenges, the interest in plant-based preservatives has been gradually declining in the past decade.

## 9. Conclusions and Future Perspective

*Mentha* species and their compounds have long been used in folk medicines and as flavoring agents. The plants and their extracts are used against digestive, nausea, fevers, headache, tumors, and skin diseases. Numerous essential oils and phytochemicals are reported from *Mentha* species, which possess different biological activities. These essential oils and their antioxidant, antidiabetic, and antimicrobial potential demonstrate that *Mentha* species could be an extraordinary source for the prevention of cardiovascular diseases. In order to utilize plant extracts to their complete application, there are several avenues that must be explored further. First, future research should focus on the modes of action of the natural compounds present in the extracts. Second, the metabolic pathways which help keep the food taste and aroma alive should be identified. These are important research questions to explore the core substances necessary for the control of diabetes and cardiovascular diseases via *Mentha* species compounds in food or in medicine. Advances in the research of medicinal plants will help in determining the quantity and quality of plant extracts required as food additives and preservatives against a specific disease. A final future potential for *Mentha* extracts does not lie in their potential medicinal values directly, but in their possible use as synergist compounds and processing mechanisms. The applications of natural antidiabetic and cardioprotective agents are likely to grow steadily in the future because of consumer demand for food containing naturally derived preservatives with good taste and aroma, such as *Mentha*.

## Figures and Tables

**Table 1:** Essential oil composition and biological activities of some *Mentha* species.

Species Name	Essential Oil	Chemical Composition	Composition (%)	Structure	Source	Activities	Reference
*M. × piperita* L.	Monoterpenoids	1-menthone	7.32–18.32	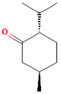	Aerial parts	Antiinflammatory, antibacterial, neuroprotective, antifatigue, and antioxidant properties	[50]
		Isomenthone	0–6.75	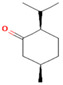	Aerial parts	Antiviral, scolicidal, immunomodulatory, antitumor, and antioxidant properties	[51]
		Menthol	18.03–58.42	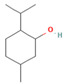	Aerial parts	Antitumor, neuroprotective, antifatigue, and antioxidant properties	[51]
		Menthyl acetate	0.72–6.89	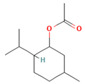	Aerial parts	Antimicrobial and flavoring agent	[50]
	Sesquiterpenes	Caryophyllene	0.05–1.54	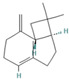	Aerial parts	Anticancer and analgesic properties	[52]
		Germacrene-D	0.63–1.89	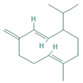	Aerial parts	Antioxidant and immunomodulatory effects	[53]
*M. longifolia* L.	Monoterpenoids	Endo-Borneol	1.12–6.02	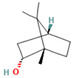	Aerial parts	Cytotoxicity and anticancer properties	[54]
		α-Terpineol	0–0.28	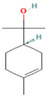	Aerial parts	Antioxidant and anti-COX-2 activity	[54]
		Isopiperitenone	0.07–0.36	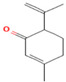	Aerial parts	Antimicrobial properties	[55]
		Carvacrol	0–1.06	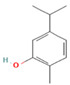	Aerial parts	Antimicrobial and Cytotoxic properties	[54]
		Cinerolon	0.08–0.25	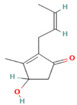	Aerial parts	Antimicrobial properties	[55]
		Cis-a-Farnescene	1.03–1.97	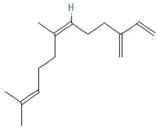	Aerial parts	Antimicrobial properties	[55]
	Sesquiterpene	Caryophyllene	2.72–7.03	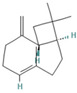	Aerial parts	Anticancer and analgesic properties	[56]
		Germacrene D	0.98–3.22	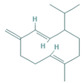	Aerial parts	Antioxidant and immunomodulatory effects	[56]
		Caryophyllene oxide	0.12–0.79	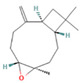	Aerial parts	Anticancer properties	[56]
*M. pulegium* L.	Oxygenated Monoterpenes	Carvone	56.1	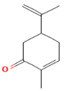	Aerial parts	Antimicrobial, antioxidant, diuretic, analgesic, and antiseptic properties	[57]
		Limonene	15.1	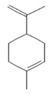	Aerial parts	Antimicrobial, antioxidant, diuretic, analgesic, and antiseptic properties	[57]
		(E)-caryophyllene	3.6	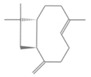	Aerial parts	Anticancer and analgesic properties	[57]
		Oleic acid	3.2	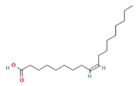	Aerial parts	Antioxidant and antimicrobial properties	[58]
		1,8-cineole	2.4	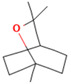	Aerial parts	Antimicrobial, antioxidant, diuretic, analgesic, and antiseptic properties	[57]
	Monoterpene	Pulegone	54.3	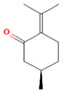	Aerial parts	Antioxidant and antimicrobial properties	[58]
*M. arvensis* L.		Menthol	30.35	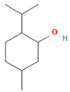	Leaf	Antiseptic, antibacterial properties, antioxidant, antimicrobial, anticancer, and antiinflammatory activities	[59]
		Menthone	20.50	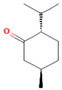	Leaf	Antiseptic, antibacterial properties, antioxidant, antimicrobial, anticancer, and antiinflammatory activities	[59]
		β-pinene	7.28	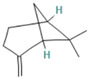	Leaf	Antimicrobial properties	[53]
		α-terpineol	7.08	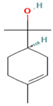	Leaf	Antiproliferative activity	[60]
		α-pinene	6.35	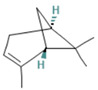	Leaf	Antiproliferative activity	[60]
		Menthofuran	5.85	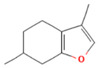	Leaf	Antioxidant, antimicrobial, cytotoxic, analgesic	[61]
		Iso-menthone	4.53	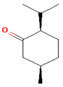	Leaf	Antiviral, scolicidal, immunomodulatory, antitumor, and antioxidant properties	[51]
		Neo-menthol	4.36	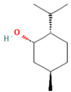	Leaf	Antioxidant properties and antimicrobial activity	[51]
		Menthyl acetate	3.26	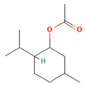	Leaf	Antimicrobial properties and flavoring agent	[50]
*M. spicata* L.	Terpenoids	Carvone	58.22	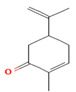	Leaf	Antimicrobial, antioxidant, diuretic, analgesic, and antiseptic properties	[57]
	Oxygenated Monoterpenes	Limonene	19.54	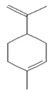	Leaf	Antimicrobial, antioxidant, diuretic, analgesic, and antiseptic properties	[57]
*M. suaveolens* L.	Terpenoids	Carvone	64.31	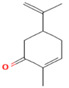	Leaf	Antimicrobial, antioxidant, diuretic, analgesic, and antiseptic properties	[57]
	Monoterpenoid	Myrcenol	5.88	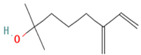	Leaf	Antioxidants, antifungal, and flavoring agents	[48]
		Terpineol	5.61	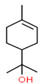	Leaf	Antimutagenic potency	[62]
		Pulegone	3.81	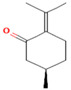	Whole plant	Antibacterial and antifungal properties	[63]
	Oxygenated Monoterpenes	Limonene	1.24	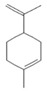	Leaf	Antidiabetic, antioxidant, and antibacterial properties	[64]
*M. aquatica* L.	Monoterpene	Pulegone	39.36	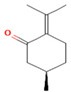	Leaves	Antioxidant and antibacterial properties	[65]
		Menthone	27.69	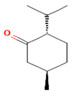	Leaves	Antioxidant and antibacterial properties	[65]
*M. virdis* L.	Oxygenated Monoterpenes	Carvone	37.26	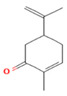	Leaves	Antioxidant, antidiabetic, dermatoprotective, antidermatophyte, and antibacterial properties	[3]
		1.8-Cineole	11.82	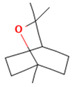	Leaves	Antioxidant, antidiabetic, dermatoprotective, antidermatophyte, and antibacterial properties	[3]
		Terpinen-4-ol	08.72	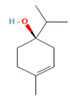	Leaves	Antioxidant, antidiabetic, dermatoprotective, antidermatophyte, and antibacterial properties	[3]

**Note**: The structures were obtained from https://pubchem.ncbi.nlm.nih.gov/ (Accessed on 15 August 2022).

**Table 2:** Antimicrobial activity of some *Mentha* species.

Species Name	Sample Used	Microorganisms	Activities	References
*M. aquatica* L.	Essential oil	*Staphylococcus aureus*, *Escherichia coli*, *Bacillus* sp., and *Candida* sp.	Showed activity against *S. aureus*, *E. coli*, and *Bacillus* sp., but no result against *Candida* sp.	[65]
*M. arvensis* L.	Ethanol extract	*Acinetobacter baumannii*	Results showed 34.5 mm inhibition use at 100 μg/mL	[85]
*M. ervine* L.	Essential oil, pulegone, isomenthone and menthone was used	*S. aureus*, *S. caprae*, *Enterococcus faecalis*, *E. faecium*, *E. hirae*, *E. coli*, *Salmonella braenderup*, *S. typhimurium*, *S. choleraesuis*, *Klebsiella pneumonia*, *Acinetobacter baumannii*, *Pseudomonas aeruginosa*, and *Listeria monocytogenes* strains	Showed variation in activity against Gram positive and Gram-negative strains, but overall inhibition activity was significant as compared to control	[86]
*M. arvensis* var. *piperascens* Malinv. ex Holmes	Essential oil	*Salmonella enteritidis*, *E. coli*, *Clostridium Perfringens*, *Campylobacter jejuni*, and *Salmonella* species	Significant antibacterial activity against all strains specifically *Salmonella* species and *C. jejuni* (inhibition zones more than 40 mm)	[87]
*M. × piperita* L.	Essential oil	*Aeromonas* spp.	High antibacterial activity was shown by the essential oils, ranging from 1.250 to 16.67 μL/mL	[88]
*M. longifolia* L.	Essential oils, Menthone, carvone Menthol, and piperitenone oxide	*S. aureus*, *E. coli*, *B. subtilis*, *Aspergillus flavus*, *Alternaria solani*, *Aspergillus niger*, *Alternaria altarnata*, *Rhizopus solani*, *Fusarium solani*, and *Rhizopus* sp.	Among these, menthol showed high antimicrobial activity, with the inhibition zone ranging from 19–33 mm	[82]
*M. itrate* Ehrh.	Essential oils	*E. coli*, *K. pneumonia*, *Salmonella typhimurium*, *Staphylococcus epidermidis*, *Streptococcus mutans*, *P. aeruginosa*, and *S. aureus*	Average activity was recorded from between 10–15 mm inhibition zone against different strains, but essential oil showed an inhibition zone of more than 15 mm against *S. epidermidis*	[89]
*M. haplocalyx* Briq.	Methanol extract	*E. coli*	Potential activity against *E. coli*	[90]
*M. pulegium* L.	Essential oil	*S. aureus*	Significant activity was observed as compared to control	[91]
*M. requienii* Bentham	Essential oil	*Aspergillus fumigatus*, *C. albicans*, *Fusarium* spp., and *Rhodotorula* sp.	Revealed sufficient inhibition against molds and yeasts, ranging from 40 mm to 70 mm	[92]

**Table 3:** Cardioprotecting effects of active constituents of *Mentha* species.

Species Name	Compounds	Cardioprotecting Effect	References
*Mentha arvensis* L.	Phenolic compounds, Menthol	Ischemic heart disease	[105]
*Mentha x piperita* L.	Aqueous extract	Antiinflammatory	[100]
*Mentha pulegium* L.	Phenolic compounds, Quercitin	Cardioprotective effects	[106]
*Mentha aquatica* L.	Menthofuran	Antiinflammatory	[107]
	D-carvone	Decrease toxicity	[118]
*Mentha canadensis* L.	Menthol	Reduce lipid peroxidation	[119]
	Menthone	Reversible cardiac depression	[119]
	Pulegone	Antiinflammatory effect	[108]
*Mentha cardiaca* J. Gerard ex Baker	Carvone	Decrease toxicity	[118]
	Limonene	Antiarrhythmic effects	[109]
*Mentha cervina* L.	Pulegone	Antiinflammatory effect	[108]
	Limonene	Antiarrhythmic properties	[109]
*Mentha diemenica* Spreng.	Pulegone	Antiinflammatory	[108]
	Menthone	Reversible cardiac depression	[119]
*Mentha longifolia* L.	Menthone	Reduce cardiac depression	[119]
	Pulegone	Antiinflammatory	[108]
	Piperitone	Induce changes in mean aortic pressure and heart rate	[110]
*Mentha pulegium* L.	Pulegone	Suppress the NLRP3 inflammasome	[120]
	Piperitone	Normalize heart rate	[110]
*Mentha spicata* L.	Carvone	Antioxidant	[121]
	Cis-carveol	Antihypersensitive, Antioxidant	[122]
	Piperitenone	Antiinflammatory	[111]
	Limonene	Antiarrhythmic properties	[109]

## Data Availability

Not applicable.
